# A Physiology-Based Model of Human Bile Acid Metabolism for Predicting Bile Acid Tissue Levels After Drug Administration in Healthy Subjects and BRIC Type 2 Patients

**DOI:** 10.3389/fphys.2019.01192

**Published:** 2019-09-27

**Authors:** Vanessa Baier, Henrik Cordes, Christoph Thiel, José V. Castell, Ulf P. Neumann, Lars M. Blank, Lars Kuepfer

**Affiliations:** ^1^Institute of Applied Microbiology (iAMB), Aachen Biology and Biotechnology (ABBt), RWTH Aachen University, Aachen, Germany; ^2^Department of Surgery, University Hospital Aachen, Aachen, Germany; ^3^Unit of Experimental Hepatology, IIS Hospital La Fe, Faculty of Medicine, University of Valencia and CIBEREHD, Valencia, Spain

**Keywords:** PBPK, computational modelling, DILI, bile acids, BRIC type 2, cholestasis

## Abstract

Drug-induced liver injury (DILI) is a matter of concern in the course of drug development and patient safety, often leading to discontinuation of drug-development programs or early withdrawal of drugs from market. Hepatocellular toxicity or impairment of bile acid (BA) metabolism, known as cholestasis, are the two clinical forms of DILI. Whole-body physiology-based modelling allows a mechanistic investigation of the physiological processes leading to cholestasis in man. Objectives of the present study were: (1) the development of a physiology-based model of the human BA metabolism, (2) population-based model validation and characterisation, and (3) the prediction and quantification of altered BA levels in special genotype subgroups and after drug administration. The developed physiology-based bile acid (PBBA) model describes the systemic BA circulation in humans and includes mechanistically relevant active and passive processes such as the hepatic synthesis, gallbladder emptying, transition through the gastrointestinal tract, reabsorption into the liver, distribution within the whole body, and excretion via urine and faeces. The kinetics of active processes were determined for the exemplary BA glycochenodeoxycholic acid (GCDCA) based on blood plasma concentration-time profiles. The robustness of our PBBA model was verified with population simulations of healthy individuals. In addition to plasma levels, the possibility to estimate BA concentrations in relevant tissues like the intracellular space of the liver enhance the mechanistic understanding of cholestasis. We analysed BA levels in various tissues of Benign Recurrent Intrahepatic Cholestasis type 2 (BRIC2) patients and our simulations suggest a higher susceptibility of BRIC2 patients toward cholestatic DILI due to BA accumulation in the liver. The effect of drugs on systemic BA levels were simulated for cyclosporine A (CsA). Our results confirmed the higher risk of DILI after CsA administration in healthy and BRIC2 patients. The presented PBBA model enhances our mechanistic understanding underlying cholestasis and drug-induced alterations of BA levels in blood and organs. The developed PBBA model might be applied in the future to anticipate potential risk of cholestasis in patients.

## Introduction

Drug-induced liver injury (DILI) places an enormous burden on health care systems worldwide. About 2–19 incidences per 100,000 habitants occur annually in Europe, with symptoms ranging from mild forms such as slightly elevated blood levels of liver enzymes to fatal clinical incidents resulting in acute liver failure (Kaplowitz, 2005; Björnsson, 2016). Due to this medical relevance, the detection of DILI at an early stage would be highly beneficial, both for a duly termination of treatment with the DILI-causing compound as well as for an early start of therapeutic interventions with curative counteragents. Manifestations of DILI can be differentiated in hepatocellular DILI, where the cellular damage of the hepatocytes dominates, in cholestatic DILI, where impaired transport functions of hepatocytes and cholangiocytes are the predominant alteration, or in a mixed type showing clinical features of both phenotypes of DILI (Hamilton et al., 2016). For the categorisation of DILI, current clinical diagnosis guidelines rely on the increase in blood plasma levels of the enzymes alanine transferase (ALT) and alkaline phosphatase (ALP). Elevated ALT levels are a general surrogate marker for hepatocellular damage as ALT is released into the blood from the cytoplasm of severely injured hepatocytes (Chalasani et al., 2014). In contrast, increased ALP levels are a specific marker for cholestasis since ALP is released from damaged cholangiocytes as a consequence of impaired bile flux in bile ducts (Vinken, 2013). Still, increased ALT and ALP levels are endpoints that only become noticeable once the liver damage has already occurred.

Ideally, biomarkers would anticipate cholestasis before the hepatic injury actually occurs in a DILI event. To achieve this goal, a mechanistic understanding of the underlying physiological alterations of bile production and transport is required. For hepatocellular DILI, a number of *in vitro* assays as well as computational models are already available allowing the analysis of drug-induced responses and alterations of intracellular metabolic pathways (Kullak-Ublick et al., 2017). Cholestatic DILI on the contrary is more complex to investigate since it originates from an altered crosstalk between liver and gastrointestinal tract at the whole-body level. The altered crosstalk results in impairment of bile acid (BA) formation and circulation. BAs are endogenous metabolites with various functions. Their detergent properties facilitate micelle formation allowing the solubilisation of lipids and thereby enabling the absorption of diet fat and fat-soluble vitamins (Jansen et al., 2017). In addition, BAs are the result of the catabolism of cholesterol and constitute a major pathway for its elimination. Because of its amphipathic nature, BAs confer the ability of bile to facilitate the excretion of lipophilic substances (Hofmann, 1999a). Furthermore, BAs function as endogenous signalling molecules in different pathways like homoeostasis control of cholesterol, energy and glucose (Houten et al., 2006).

BA metabolism is a nearly closed circuit including *de novo* synthesis, transformation, diffusion and intestinal reabsorption as well as multiple active transport processes. Within the body, BAs undergo continuous enterohepatic circulation connecting liver and gastrointestinal tract through the gut-liver axis. The total BA pool comprises a broad variety of conjugated and unconjugated BA species. BAs are synthesised *de novo* by hepatocytes and conjugated with glycine and taurine before leaving the liver as primary bile acids. Following its synthesis, BAs are actively secreted by hepatocytes. In hepatocytes, they can be transported either to bile canaliculi (apical) or to the liver sinusoids (basolateral). BAs secreted into bile canaliculi and bile ducts accumulate in the gallbladder. From there, they are released into the luminal space of the duodenum, and subsequently metabolised by the microbial gut flora secondary bile acids. to Secondary BAs are absorbed from the intestinal lumen by gut enterocytes. From there they are secreted to either the gut lumen (apical) or to the blood capillary vessels (basolateral). Those BAs secreted basolaterally reach again the liver via portal vein (enterohepatic circulation), and thereafter enter the vascular circulation and eventually reach other tissues. Notably, these transporter-mediated processes are key steps in enterohepatic circulation which have a significant impact on dynamics and mass distribution of the BA pool.

Due to an effective recycling, only around 5% of the BA pool is lost over 24 h mainly via faeces (Houten et al., 2006). Hence, the turnover of BAs is a systemic process that involves different tissues and active enzymatic and transport processes. An impairment of for example canalicular BA transporters, such as Na^+^-taurocholate co-transporting polypeptide (NTCP), multidrug resistance protein 1/3 (MDR1/3), multidrug resistance-associated protein 2 (MDR2) and bile salt export pump (BSEP), results in the accumulation of BAs in the liver or other tissues, with potential toxic consequences (Jackson et al., 2016; Wagner and Trauner, 2016; Castro and Pereira Rodrigues, 2017).

Such an accumulation of BAs in the liver or other tissues leads to the clinical symptoms of cholestasis (pruritus and jaundice, when bilirubin transport is also impaired). In the beginning of DILI pathogenesis, plasma BA levels start to increase before the cellular damage finally occurs. Hence, the rise of BA concentration in blood, along with their composition pattern would be an ideal early biomarker for cholestasis from a medical perspective. Recent improvements in analytical methods facilitate a fine-tuned analysis of different BA species for a differential diagnosis of DILI (García-Cañaveras et al., 2012, 2014). In clinical practice, these analytics are, however, still not applicable as a routine standard methodology. In addition, BA composition is influenced by the sampling site and plasma profiles might not be representative for the concentrations of a species present in tissues. Furthermore, it is difficult to assess the relevance of bile acid profiles in the various types of cholestasis (Eggink et al., 2018). This is even more relevant in the case of *in vitro* experiments where bile acid circulation among different tissues cannot be modelled appropriately. *In vitro*, such a scenario can only partially be achieved in an organotypic microenvironments as e.g., in sandwich or spheroidal microtissue cultures (Bell et al., 2016), or in much more complex experimental settings that incorporate microfluidics to reproduce the interplay with other organs (Kimura et al., 2018; Sudo, 2019). Therefore, even advanced assays can only focus on limited aspects of cholestasis, like the BA uptake and excretion by liver parenchymal cells and potential interferences of drugs with BA hepatic transporters.

Computational modelling stands as a tool that can contribute to a mechanistic understanding of the interplay of the various physiological processes underlying the BA metabolism. In the case of cholestasis, such computational models, which ideally should be knowledge-driven and physiology-based, have to account for the enterohepatic circulation of BAs as well as their accumulation in different tissues. Computational models may be used specifically to simulate physiological concentration profiles in sites that are experimentally not accessible *in vivo*, such as for example the intracellular space of different organs. In addition, computational models may help to integrate the existing knowledge in a mathematical representation to identify gaps in the current understanding of a physiological or pathological phenomenon. Likewise, they may be used to pinpoint targeted screening biomarkers for the emergence of cholestasis.

**FIGURE 1:**
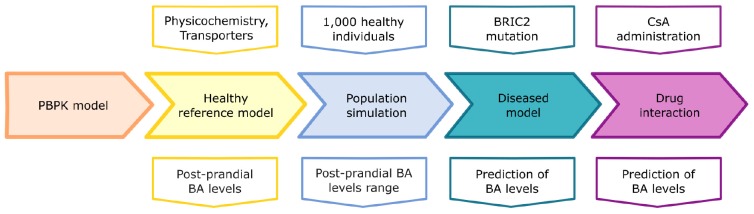
Study workflow. The five steps of model development are depicted: basic PBPK model, healthy reference model, population simulation, diseased model for BRIC, and drug interaction with Cyclosporine A (CsA). The upper row of boxes depicts the inputs for the different model stages (middle row). The lower row depicts the outputs of the model simulations.

In this study, we present a physiology-based model of bile acid metabolism at the whole-body level based on physiology-based pharmacokinetic (PBPK) modelling (Kuepfer et al., 2016). Our model describes the systemic distribution and enterohepatic circulation of glycochenodeoxycholic acid (GCDCA) as an exemplary BA. Besides the passive diffusion and distribution processes, the model includes the active processes of synthesis, transport, distribution, and excretion of GCDCA. We validated the computational physiology-based bile acid (PBBA) model with time-concentration profiles from healthy individuals. Subsequently, the PBBA model was used to analyse aberrant states of bile acid metabolism as they occur in cholestasis. First, the model was used to analyse shifts in BA levels due to a genetic predisposition for cholestasis in BRIC2 patients (BRIC2: benign recurrent intrahepatic cholestasis type 2). These patients are mostly asymptomatic but may develop symptoms of cholestasis following medical incidents as for example drug intake. Secondly, we applied the model to examine cholestasis induced by cyclosporine A (CsA) which is known to competitively inhibit canalicular BA transporters, as a representative case of drug-induced cholestasis. This overall workflow is depicted in Figure 1.

## Materials and Methods

### PBPK Modelling

Physiology-based pharmacokinetic models mathematically describe the physiological processes underlying absorption, distribution, metabolism, and excretion (ADME) of compounds such as xenobiotics or endogenous molecules within the body of an organism at a large level of physiological detail (Kuepfer et al., 2016). PBPK models include all major organs of the organism, such as the liver, heart, or kidney. The organs are further subdivided into different compartments such as plasma, red blood cells, interstitial and intracellular space. Since PBPK models are knowledge-based, most parameters describing the anatomy or physiology of the body for example organ volumes, surface areas, or blood perfusion rates are taken from curated data collections, usually provided within the PBPK software. The different organs are interconnected through the vascular circulation. Such a high level of detail of the organism physiology allows a mechanistic representation of complex biological systems and phenomena as well as the individualisation of patient models through the consideration of specific phenotypes or other physiological characteristics. Physiologically relevant and tissue-specific active ADME processes like enzymatic metabolism and transport can also be considered in PBPK models. Tissue-specific gene expression data can be integrated as a surrogate for enzyme and transport protein levels in active processes (Meyer et al., 2012). Besides physiological and anthropometric information of the modelled organism, substance-specific physicochemical parameters like the molecular weight, solubility, or lipophilicity are used as input parameters during PBPK model development. In particular, these values are used to calculate passive diffusion processes across membranes or organ-plasma partitioning coefficients in the distribution models typically underlying PBPK models. The Open Systems Pharmacology (OSP) platform (MoBi^®^ and PK-Sim^®^) was used for PBBA model development. The latest versions of PK-Sim^®^ and MoBi^®^ are freely available under the GPLv2 License^1^.

### Model Building

**FIGURE 2:**
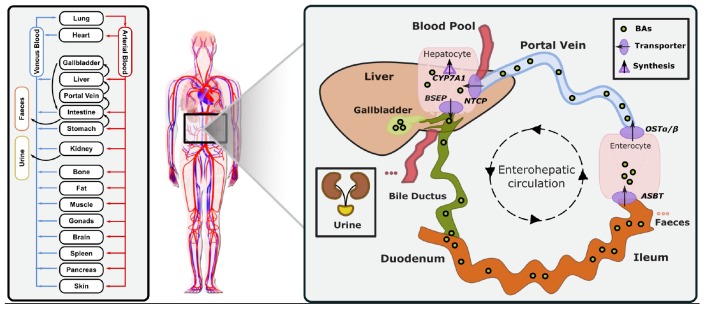
Physiology-based bile acid model (PBBA). Building on a PBPK model of the bile acid GCDCA, biosynthesis via CYP7A1 in the liver, active transport processes via BSEP, ASBT, OSTα/β, and NTCP, faecal and renal excretion were additionally included. GCDCA is stored in gallbladder and partially secreted directly into the duodenum and is reabsorbed along the intestine (enterohepatic circulation). Emptying of gallbladder is triggered by food intake.

The reference model of a healthy average individual includes synthesis, circulation and excretion of an exemplary BA (Figure 2). In our study, GCDCA was chosen since it is the most abundant BA accounting for about 20% of the total human BA pool (Bathena et al., 2013). This enabled us to reduce model complexity to the key physiological processes and improve identifiability of the free model parameters. In addition, the consideration of a lumped pool by using a single exemplary BA species allowed the integration of heterogeneous literature data that analyse different BA species. Following best practice guidelines for PBPK model building, physicochemical parameters of GCDCA like molecular weight, solubility, lipophilicity (logP), and plasma-protein binding (fraction unbound) (Table 1) were used to parametrise the compound properties of the PBPK model for small molecules. Thus, passive transport processes as well as organ-plasma partitioning can be directly calculated using an appropriate distribution model.

**TABLE 1:** Physico-chemical parameters of bile salt GCDCA.

**Parameter**	**Value**	**References**
logP	2.12	Roda et al., 1990
Fraction unbound	0.01	Roda et al., 1982
Solubility [mg/l]	100,000	Hofmann, 1999a
Molecular weight [g/mol]	449.62	Law et al., 2014
pKa	3.77	Law et al., 2014

To compensate for the daily loss of BAs, a continuous synthesis reaction was introduced to the model. This formation of GDCDA is represented by a constant synthesis in the intracellular space of the liver. *In vivo*, this synthesis rate accounts for cytochrome P450-mediated oxidation of cholesterol and subsequent conjugation with glycine within the liver (Kullak-Ublick et al., 2004; Martinot et al., 2017). In total, four active transport processes were included in the PBBA model: (1) The bile salt excretion pump (BSEP) on the apical membrane of hepatocytes, (2) the NTCP on the basolateral membrane of hepatocytes, (3) the human ileal bile acid transporter (ASBT) apically in the ileum mucosa, and (4) the organic solute and steroid transporter (OSTα/β) basolaterally in the ileum mucosa (Kullak-Ublick et al., 2004; Rao et al., 2008). A fraction of 65% of biliary excreted BAs was assumed to be stored in the gallbladder while the remaining fraction is directly secreted to the duodenum (Hofmann, 1999a). Gallbladder emptying is triggered by meal ingestions. In all simulations, three meals over 24 h representing breakfast, lunch and dinner were considered. Such emptying processes can be modelled via inbuilt plug-ins of the OSP Suite and their values were adapted to fit the experimental data (Table 2). To close the overall mass balance, faecal and renal excretion of GCDCA were implemented in the model by passive transport and active clearance, respectively (Table 3). Altogether, the initial PBPK model of GCDCA structurally describes continuous BA synthesis as well as enterohepatic circulation through the liver and the gastro-intestinal tract including re-absorption from the ileum.

**TABLE 2:** Values of standard PBPK model parameters.

**Parameter**	**Value**	**Start value**
Body weight [kg]	73	Fixed
Age [years]	30	Fixed
Height [m]	1.76	Fixed
Distribution model	Rodgers & Rowland	Rodgers & Rowland
GB Volume [l]	0.05	Fixed^1^
Refilling time [min]	147.8	419
Emptying half-life [min]	69.98	69.98

*^1^van Erpecum et al., 1992.*

**TABLE 3:** Values of transport processes, synthesis, and inhibition for the PBBA model parameters.

**Parameter**	**Value**	**Start value**
*K*_m_ (BSEP) [μmol/l]	5	4^1^
*K*_m_ (ASBT) [μmol/l]	0.5	50
*K*_m_ (NTCP) [μmol/l]	1	6^1^
*K*_m_ (OSTα/β) [μmol/l]	7.5	50
*k*_cat_ (BSEP) [1/min]	300	100
*k*_cat_ (ASBT) [1/min]	5	100
*k*_cat_ (NTCP) [1/min]	125	100
*k*_cat_ (OSTα/β) [1/min]	9000	1000
Synthesis rate [μmol/min]	0.78	Fixed^1^
Renal excretion [μmol/l/min]	981.30	100^2^
*K*_i_ (CsA) [μmol/l]	2	2^3^

*^1^Kullak-Ublick et al., 2004; ^2^Bathena et al., 2015; ^3^Böhme et al., 1993.*

Next, uninformed model parameters were identified in order to accurately describe the dynamics of the BA metabolism in a healthy reference individual. Importantly, only a limited set of modelling parameters had to be considered since the model relies on large datasets of physiological and physicochemical information as provided by the underlying PBPK model. The basic PBBA model mainly describes GCDCA with its passive distribution and the transport molecules with their transport processes and was established within PK-Sim^®^. The additional endogenous processes, i.e., BA synthesis and gallbladder emptying events, were implemented in MoBi^®^. The model along with a technical building instruction are available from the Supplementary Material.

For the population simulations a virtual population of 1,000 healthy individuals with varied anthropometric properties (Age: 20–60 years, females: 50%, BMI: 19–25 kg/m^2^) and reference concentrations for all transporters was constructed in PK-Sim^®^. Up to 10% variation was allowed for the transporters’ abundance. Population simulations and model analyses were performed in Matlab with standard boxplot function (Version 8.5.1.281278; The MathWorks Inc., Natick, MA, United States). The population parameters are available from the Supplementary Material.

### Competitive Inhibition of BSEP Transport by Cyclosporine A

A PBPK model of CsA was previously developed with PK-Sim^®^ (Thiel et al., 2017b) and was integrated in the PBBA model to simulate the effects of CsA on BA levels. Additionally, a term describing the competitive inhibition by the drug on BSEP transport kinetics was introduced to the integrated model as follows:

$$v_{\mathrm{BSEP}}=v_{\max}\times \frac{\left[S\right]}{K_{m,\mathrm{app}}+\left[S\right]}\,\mathrm{with}\,K_{m,\mathrm{app}}=K_{m}\times \left(1+\frac{\left[I\right]}{K_{i}}\right),$$

Where [*S*] is the concentration of BSEP substrate GCDCA, *K*_m,app_ is the apparent *K*_m_ as defined as above, [*I*] is the concentration of the inhibitor CsA, *K*_i_ is the inhibitor’s dissociation constant, and *K*_m_ is the Michaelis Menten constant (Berg et al., 2018).

### Experimental Data

Consolidated experimental data from literature, describing BA levels in the blood plasma of healthy individuals, were used for parameter estimation and model validation. BA plasma levels under fasting conditions were used to identify the basal level of systemic BAs (Bathena et al., 2013).

In addition, results from various studies measuring postprandial plasma BA profiles after three subsequent meals in healthy male individuals (Hepner and Demers, 1977), healthy woman (Angelin and Björkhem, 1977), pregnant woman, and diseased volunteers (Schalm et al., 1978) were used to identify the system dynamics of circulating BA levels in the human body. Furthermore, we used another set of published experimental data, not used for parameter identification, to validate our model predictions and to additionally assess the variability of individual BA blood plasma levels (LaRusso et al., 1978; Ponz de Leon et al., 1978; Galeazzi et al., 1980; Gälman et al., 2005; Salemans et al., 2009). If necessary, experimental plasma BA data were extracted from the original publications with the web-based WebPlotDigitizer tool (version 3.9; Ankit Rohatgi, Austin, TX, United States, freely available under the GPLv3 License)^2^.

### Data Normalisation

Notably, the various studies measured different BA conjugates, thus we normalised the data. In the study of Hepner and Demers (1977) glycine conjugates of cholic acid (CA), chenodeoxycholic acid (CDCA), deoxycholic acid (CDA) and sulpholithocholic acid (LCA) were identified, as such representing only a subset of the complete BA pool. Another study (Schalm et al., 1978), investigated postprandial plasma BA profiles in five healthy as well as in pregnant and diseased volunteers and measured chenyl- and cholyl- conjugates. Whereas yet others (Angelin and Björkhem, 1977) measured postprandial plasma BA profiles in five healthy women and quantified CA, CDCA, and DCA without amidation and sulphation. The measured BA species vary considerably among the different studies, and we normalised the postprandial BA profiles to allow a comparison of the different data sources. Therefore, a percentage scaling factor was calculated from literature for scaling all datasets to the fraction of summed conjugated cholic, chenodeoxycholic, and deoxycholic acid as far as the study description allowed (Supplementary Table S1 and Supplementary Figure S1; Bathena et al., 2013) by the following formula *y*_n_ = *y*_old_ × scaling factor.

### Goodness of Fit

To quantify how well the model describes the data, four different measures were used:

(1) *k-fold* deviation with *k* ∈ {2,3,4}, to quantify the percentage of observed data lying within a given deviation.
(2) Root-mean-square deviation (RMSD) according to the following formula:
  $$\mathrm{RMSD}=\frac{\sum_{i=0}^{n}{\left({\mathrm{obs}}_{i}-{\mathrm{pred}}_{i}\right)}^{2}}{n}$$
(3) Normalised root-mean-square deviation (NRMSD) according to the following formula:
  $$\mathrm{NRMSD}=\frac{\mathrm{RMSD}}{\max\,\left(\mathrm{obs}\right)-\min\,\left(\mathrm{obs}\right)}$$
(4) *R*^2^ according to the following formula:
  $$R^{2}=\frac{\sum_{i=0}^{n}{\left({\mathrm{obs}}_{i}-{\mathrm{pred}}_{i}\right)}^{2}}{\sum_{i=0}^{n}{\left({\mathrm{obs}}_{i}-\bar{\mathrm{obs}}\right)}^{2}}$$

where *n* is the number of data points and $\bar{o\,b\,s}$ describes the mean of the observed datapoints.

## Results

### Physiology-Based Model of Bile Acid Metabolism

**FIGURE 3:**
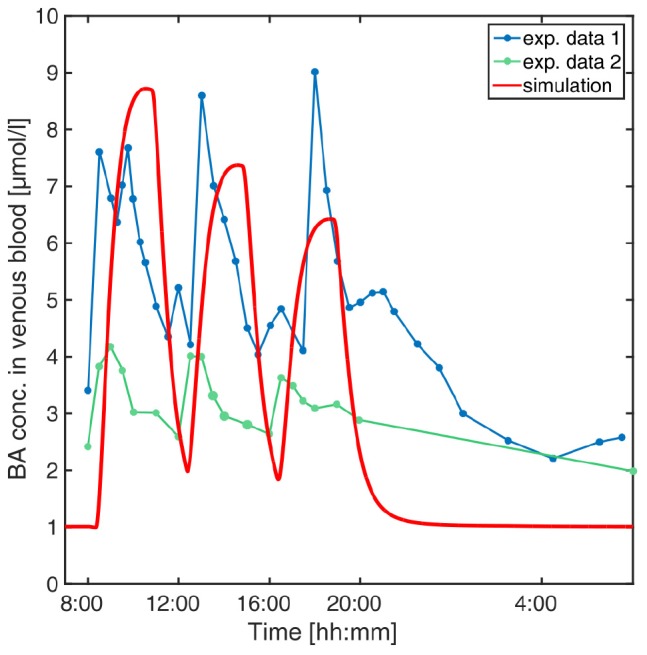
Simulation of venous blood plasma BA levels in a human reference individual. The PBBA model was simulated with three meals per day given at 8, 12, and 16 o’clock and simulated BA concentrations in venous plasma (red solid curve) are compared with reported values from Hepner and Demers (1977) (exp. data 1, dark blue points connected by dashed line) and Ponz de Leon et al. (1978) (exp. data 2, green points connected by dashed lines).

A computational physiology-based model describing the distribution and enterohepatic circulation of an exemplary bile acid at the whole-body scale in an average healthy individual has been developed. The overall workflow of the study in terms of model development and subsequent analyses is presented in Figure 1. The reference model of BA metabolism in healthy individuals was developed based on physicochemical properties of GCDCA as an exemplary BA and the known physiological processes that take place during enterohepatic circulation. BA synthesis, renal and faeces excretion, passive diffusion, gallbladder emptying, and four active transporters (BSEP, NTCP, ASBT, and OSTα) were implemented (Figure 2). Subsequently, a virtual population of 1,000 individuals was created to assess the variability in post-prandial BA levels. Also, the impact of the BRIC2 and progressive familiar intrahepatic cholestasis type 2 (PFIC2) mutations as cause of DILI predisposition and CsA administration on BA levels were analysed. A detailed description of the model is given in the Methods section. Measurements of basal and postprandial BA concentration levels from literature were used to evaluate the agreement of the computational simulations with the scaled experimental data (Hepner and Demers, 1977; Ponz de Leon et al., 1978; Schalm et al., 1978). The comparison between the simulated kinetics of BA levels over 24 h with reported data is shown in Figure 3. Experimental data of post-prandial BA profiles from two studies are shown, in which healthy individuals were fasted overnight and given three meals at 8:00, 12:00, and 16:00 h. Following parameter identification, the model could describe the plasma BA dynamics well despite a significant level of variability in the experimental data. The peak concentrations as well as the corresponding postprandial levels and the dynamics of gallbladder emptying are met with sufficient accuracy (Figure 3 and Table 4). We next verified whether several physiological reference measurements of the BA metabolism such as total BA pool size, cycling times and concentrations in various compartments could be described by the model. Hence, a series of clinical parameters were retrieved from the scientific literature and used for fitting and comparing to corresponding values calculated from the simulation results (Table 5). Even though the model is an open system with a complex dynamic behaviour, a good agreement between literature values and simulation outcomes was achieved. The accordance of physiological reference values represents a strong indication for a good overall model quality in terms of both, mass balance and dynamics (Figure 3, Tables 4, 5 and Supplementary Figure S2).

**TABLE 4:** Goodness of fit of the PBBA model.

**Parameter**	**2-fold deviation**	**3-fold deviation**	**4-fold deviation**	**RMSD**	**NRMSD**	**R^2**
Value	0.64	0.88	0.97	6.21	0.85	−0.41

**TABLE 5:** Physiological reference measurements.

**Parameter**	**Literature**	**Model**	**References**
BA conc. in venous blood [μmol/l]	[0.9, 8.4]	[1.68, 8.91]	Var. Sources (see M&M)
BA conc. in portal vein blood [μmol/l]	[2.8, 33.2]	[3.95, 34.5]	Angelin et al., 1982
Faecal excretion rate [μmol/min]	0.72^∗^	0.72	Dancygier, 2003
BA pool size [μmol]	[4250, 6672]^∗^	5697.69	Beuers et al., 1992; Bisschop et al., 2004
Avg. secretion rate per meal [mmol/h]	5	∼1.2	Hofmann, 1999a
BA conc in gallbladder [μmol/l]	[3000; 100,000]	[25;2800;5000]	Hofmann, 1999a; Jansen et al., 2017
BA conc. in intestinal lumen [μmol/l]	[2000, 10,000]	[75, 5175.02]	Hofmann, 1999a; Hamilton et al., 2016
BA conc. in liver cells [μmol/l]	1-2;<3	[0.23, 1.07]	Hofmann, 1999a, b

*Values with (^∗^) were converted to model units.*

### Population Simulation of the PBBA Model

**FIGURE 4:**
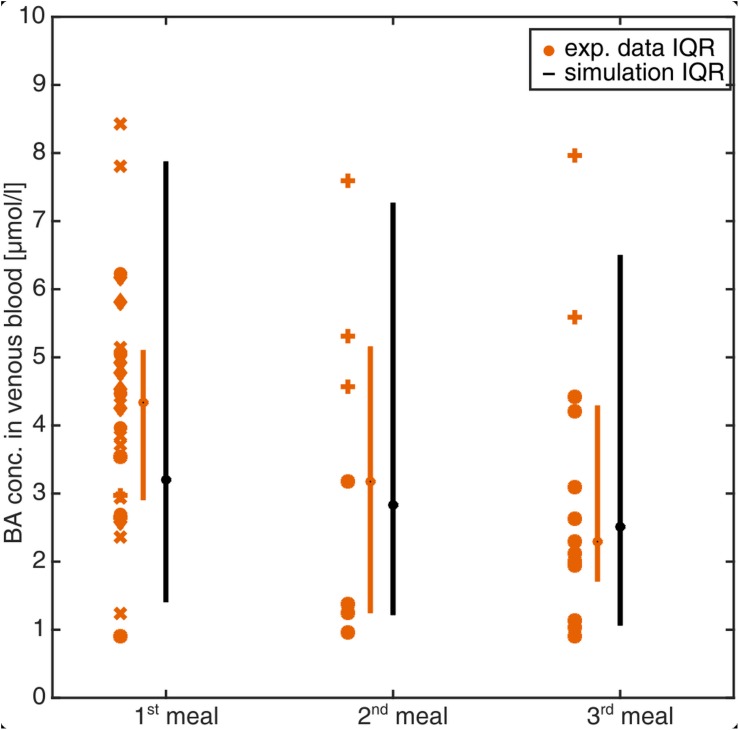
Simulation of venous blood plasma BA levels in a virtual healthy population of 1,000 individuals. BA levels were assigned to the meal after which they were measured or simulated. Symbols represent BA plasma measurements from experimental studies [o= (Angelin and Björkhem, 1977), + = (Galeazzi et al., 1980), ^∗^ = (Gälman et al., 2005), x = (Salemans et al., 2009), ♢ = (Ponz de Leon et al., 1978)]. Bars (compact boxes) represent the interquartile range of the experimental data (orange) and the simulation (black) with the median marked as dot on the box.

The developed PBBA model describes BA profiles in an average adult individual. This is a far-reaching assumption given the significant inter-individual variability in the clinical data (Figure 3). To test the robustness of the PBBA model, a population simulation was additionally performed. For this, a virtual population of 1,000 individuals was created within PK-Sim with the parameters given in the see section “Materials and Methods”. To account for variability in the transporter expression, which are not part of the PK-Sim database for populations, a 10% variability was assumed. The simulations were run to steady state and Figure 4 shows the BA levels per meal from the population simulation and experimental data (see section “Materials and Methods”). Data, both from literature and the simulations were assigned to a first, second, and, third meal whenever possible. Single BA measurement in plasma (one symbol per study) as well as boxes condensed to bars indicating the 25th and 75th percentiles (IQR) for the experimental data and the simulations are shown. The median BA concentration per each meal decreases over daytime in both, the experimental data and the simulations. The predicted population variability was in a to-be-expected physiological range and matched the experimentally measured BA values (80 and 100% of observed data within the IQR and the 95th and 5th percentiles of observed data, respectively).

The comparison of the PBBA model with various physiological reference measurements (Table 5) as well as clinical data sets (Figure 4) is a strong indication for the overall correctness of the model for healthy reference individuals. This positive validation of our computer model gives confidence for further predictions and investigations.

### Diseased Model of Benign Recurrent Intrahepatic Cholestasis Type 2 (BRIC2)

A variety of clinical cases of cholestasis result from inborn mutations in humans (Pauli-Magnus et al., 2005). Depending on the affected protein and the locus of mutation, different types and severity of cholestasis may emerge. It is known that carriers of PFIC2 or BRIC2 have a higher risk of encountering cholestasis as a consequence of other diseases or drug therapies (Srivastava, 2014). Both the severe PFIC2 and the milder BRIC2 are caused by polymorphisms of the BSEP-coding gene which lead to an impaired function of the encoded protein. As a result, PFIC2 patients usually experience an early onset of cholestasis in their lifetime and often need early liver transplantations. The BRIC2 mutations are usually less severe such that a basal functionality of BSEP remains. However, affected patients have clinical episodes of cholestasis during their lifetime and slightly elevated basal BA plasma levels (Ermis et al., 2010; Zellos et al., 2012; Hayashi et al., 2016).

**FIGURE 5:**
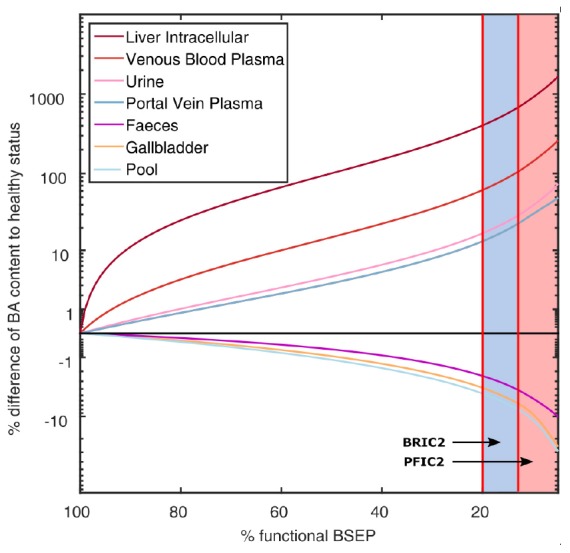
Simulation of BA levels in various compartments with decreasing BSEP function. The mean difference (in% of reference) of BA content in various compartments as well as the total BA pool are plotted over decreasing BSEP function. The reported ranges of BSEP functionality in BRIC2 patients with 20–13% and in PFIC2 patients with <13% are marked in blue and red, respectively. BA content in faeces, gallbladder, and the total pool decrease with decreasing BSEP function, while BA in liver cells, venous blood, portal vein blood, and urine increase.

Based on the PBBA model developed for healthy individuals, we simulated the effect of PFIC2 and BRIC2 on systemic BA levels by decreasing the transporter activity in this genotype subgroup. For BRIC2 patients, we reduced the BSEP *k*_cat_ from 100% to 20–13% of the original BSEP transporter activity to account for the remaining functionality. For the PFIC2 genotype, the transporter activity of BSEP was further reduced to 5% (Noe et al., 2005). The simulation results show the relative differences of BA amount in various enterohepatic compartments after simulating the gradual loss of BSEP function (Figure 5). While the downstream compartments of the liver including the gallbladder, intestinal tract (not shown), and faeces contain less BAs, the upstream compartments portal vein, venous blood, and urine contain higher amounts of BAs compared to simulation results with 100% BSEP function. The range of BSEP function in BRIC2 individuals is indicated in blue and simulations show that individuals have up to doubled BA levels in blood and up to six-fold increase in the liver cells (Figure 5).

### Drug Interaction With Cyclosporine A

**FIGURE 6:**
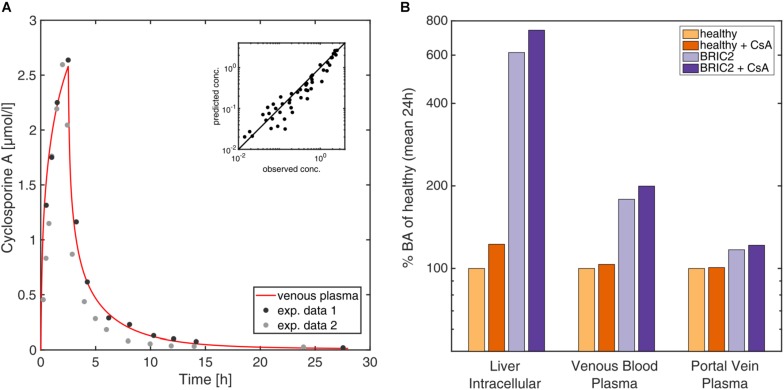
**(A)** Time-concentration profile of CsA in venous blood after oral intake of 4 mg/kg. Observed (Aweeka et al., 1994; Thiel et al., 2017b) versus simulated concentrations of CsA levels in venous blood plasma after intravenous and oral administration of 4 mg/kg and 10 mg/kg. **(B)** 7 Simulation of the coupled CsA PBPK and the PBBA model. Simulated mean BA (in% of healthy reference) of the reference model, the BRIC2 and the CsA-coupled models in the intracellular space of the liver, venous blood plasma, and portal vein plasma are shown.

As another clinical scenario we modelled the influence that CsA administration could exert on BA levels. CsA is known to induce cholestatic DILI with different degrees of severity by influencing gene expression of liver enzymes and transporters, but also inhibiting transport processes of BSEP competitively. We incorporated in the PBBA model a previously published PBPK model of CsA (Thiel et al., 2017b) to investigate the potential impact of the drug on BA levels. Drug-drug interaction models are a frequent application in PBPK modelling (Thiel et al., 2017a), however, it should be noted that in the present case, the coupled model simultaneously describes the disposition of endogenous BA species as well as the pharmacokinetics (PK) of an exogenous drug. The PBPK model for CsA has been carefully validated before with different PK data for intravenous and oral administration (Thiel et al., 2017b; Figure 6). The inhibition of CsA on BSEP was integrated by the introduction of a competitive inhibition term for the BSEP kinetics (see section “Materials and Methods”). Simulations were performed for healthy individuals as well as BRIC2 patients. Figure 6A shows the CsA levels after a bi-daily intravenous dose of 2 mg/kg CsA in venous blood and liver cells. The simulations show mildly elevated BA levels in venous and portal vein blood in healthy individuals (Figure 6B). A more pronounced effect is observed in the liver, where BA levels increase about 22% compared to the untreated case. After CsA administration in BRIC2 patients, the model anticipates BA levels increase up to eight-fold, relative to healthy reference individual (Figure 6B). These results suggest that even routine medical treatments may increase BA plasma concentrations in BRIC2 patients to levels clearly above normality indicating a potentially cholestatic effect.

## Discussion and Conclusion

Distribution and accumulation of BAs in blood plasma and tissues is a direct clinical biomarker for cholestasis. However, assessment of BAs accumulation within different tissues is infeasible due to technical and ethical limitations. Therefore, a truly comprehensive picture of BA distribution and metabolism cannot be achieved from clinical measurements alone. Enterohepatic circulation of BAs, a systemic process which involves multiple consecutive and fine-tuned steps in different organs, adds even more complexity and variability. Altogether, the causes of altered BA metabolism and accumulation in tissues are difficult to precisely monitor in individual patients, what significantly limits the usage of BAs measurement for diagnostic profiling. A computational model capable of quantitatively describing BA concentration in body fluids and tissues could be a valuable tool to better understand and interpret the alterations of the systemic BA distribution and metabolism. Such a mechanism-based computer model could be helpful to identify novel (early) markers for cholestasis in clinical practice. Moreover, it could be useful to understand the underlying mechanisms of cholestasis, anticipate toxic effects and envisage clinical strategies to improve patient’s recovery once the first cholestasis DILI symptoms have been recognised.

The computational whole-body PBBA model described allows the simulation of BA exposure in blood plasma and different tissues. The main processes of BA metabolism such as synthesis, excretion, and enterohepatic circulation are mechanistically included in the model at a large level of physiological detail based on the underlying PBPK model structure. Likewise, as the model builds on the well-established PBPK framework, organ-plasma partitioning is explicitly represented for different tissues throughout the body. It should be noted that as such the model is similar to a typical PBPK model for xenobiotic drugs even though distribution and excretion of an endogenous compound are considered here. This similarity is a particular advantage of our approach since the basic PBPK model already includes a detailed physiological representation of the gastro-intestinal tract involving several segments to quantitatively describe dissolution of tablets (Thelen et al., 2011). This is of outmost importance to physiologically describe re-absorption of BAs from the gut lumen during enterohepatic circulation. With this model intracellular concentrations, e.g., in the liver, are directly available. Additionally, concentrations in other off-target tissues such as skin or brain may also be simulated to evaluate the risk of complications like pruritus.

We initially parametrised the presented PBBA model based on a comprehensive set of plasma BA data. Subsequent model validations were conducted with independent data sets not used during model establishment. We showed that the PBBA model is capable of reproducing the dynamic postprandial BA levels in healthy individuals, as well as to simulate BA levels for different clinical disease cases of inborn errors and for inhibitory interactions after drug-administration. However, a perfect correlation of the model with the observed data cannot be expected due to multiple factors. On one hand, the BA lumping introduces some bias since the kinetics of different BA species vary considerably (Hepner and Demers, 1977). But modelling of different BA species with their kinetics is hampered by the limited and sometimes conflicting data what is displayed in Figure 3. On the other hand, the general variability in BA blood levels is high even under healthy conditions. Therefore, we decided to further validate the model to additional available observed concentrations and rates (Table 5). Since intrinsically, the simulation of one average individual cannot cover such inter-individual variability a population simulation was performed to confirm the model’s performance (80% of observed data within IQR, Figure 4).

The genotype-specific functionality of BSEP transporter was simulated and confirmed the predisposition of the BRIC2 subgroup toward drug-induced cholestasis by elevated BA levels in blood and in liver. The lack of clinical data renders it hard to validate the simulations, but the physiology-based mechanistic background integrates as much knowledge as we have. Therefore, our predictions are the closest quantitative guess for inaccessible but critical compartments like the liver cells. Additionally, the overall model behaved in a consistent and physiologically expected manner, indicating the appropriateness of the assumptions, equations and the restrictions self-imposed in the course of its mathematical development. The simulation of CsA administration to this patient group anticipated a large increase of the BA levels in these patients, which should warn the clinician about increasing the risk of cholestasis. Here, computational modelling allowed the quantitative estimation of tissue-specific BA exposure which is not accessible clinically. The computational PBBA model allowed a systematic consideration of different degrees of BSEP activity in BRIC2 and PFIC2 patients, which could otherwise not have been analysed. In addition, the developed PBBA model is not data-driven but rather knowledge-based, since a lot of prior physiological information is included in the underlying PBPK model. For this reason, it is also possible to extrapolate the model to consider specific questions or hypotheses like functional changes or alterations in environmental conditions, which have not been explicitly considered during model establishment itself. This has been done with PBPK models in other contexts like paediatric scaling. Willmann et al. (2014) Hence, the PBBA model presented here seems particularly well suited to simulate scenarios that can take place in patients with impaired BA transport based on the reference PBBA model of healthy reference individuals.

The first attempts to mathematically model the BA metabolism were published in the early 1980s (Hofmann et al., 1983; Cravetto et al., 1988). These models were detailed in the description of the BA species and the enterohepatic circulation but lacked mechanistic knowledge regarding the whole-body physiology and relevant transporters. Recent models make use of a simplified representation of the body physiology and do not include organs or their sub-compartments such as intracellular or interstitial space (Woodhead et al., 2014; Sips et al., 2018), as such potentially limiting the quantification of specific tissue concentration profiles. Moreover, such models are mostly data-driven limiting their translation to new indications, patient subgroups or clinical scenarios such as BRIC2 and PFIC2 patients, which were explicitly considered in this study. Since our focus lies on the basic physiological mechanisms of cholestasis development, none of the general yet secondary clinical biomarkers like ALP have been considered in our model (Woodhead et al., 2014; Longo et al., 2016). Instead, we aimed toward a description of the actual defect and not the indirect consequences of the tissue damage induced by accumulated BA. In contrast to data-driven approaches (Sips et al., 2018), the presented PBBA model is knowledge-based and relies largely on prior curated information explicitly included in the originating PBPK model. This increases the reliability of both the identified parameters and the model-based analyses.

The PBBA model might also help to explain the causalities in idiosyncratic cases of DILI such as genetic or physiological predisposition of individual patients in the future. Functional consequences of kinetic alterations such as different genotypes or diseases can be mechanistically represented in physiology-based modelling (Lippert et al., 2012; Cordes et al., 2016) allowing, for example, to describe cases of DILI beyond intrinsic, i.e., predictable dose-relations. Since PBPK models allow the inclusion of patient specific physiological information, the PBBA model might be used to analyse cases of idiosyncratic drug-induced cholestasis. In particular, the model allows the simulation of individual drug exposure in off-target tissues as a consequence of a patient’s anatomy, physiology, lifestyle, gender or age. In addition, the used PBPK framework can be used to translate model predictions from the current human PBBA model to other species like mice to support model-based experimental design (Thiel et al., 2015).

The current mathematical formulation of this model has, however, a certain number of shortcomings. The population simulation showed that the simulated variability is higher than the recorded one. This deviation is likely to be overcome by adjusting the BAs synthesis rate to the liver sizes instead of assuming a fixed liver volume. Basolateral transport processes for BA excretion from the hepatocytes were neglected since it is difficult to catch because only a net transport rate into the hepatocytes can be reliably identified. For this reason, counteracting processes which could potentially reduce the effect of BSEP functional impairment in BRIC and PFIC2 patients have not been considered so far (Figure 5). In this version of the PBBA, we only considered GCDCA as a surrogate BA. However, it is known that different BA species do not have the same kinetics (Setchell et al., 1997). Therefore, this approach may introduce a systemic error leading to a reduced agreement of the model with the experimental data. It can also be argued that, the smaller peaks secondary to the main meal peaks (Figure 3) are not reflected by the model. This is probably due to not having included the different BA species which may show different dynamics. Despite these drawbacks, it should be recognised that the model is still capable of describing the global behaviour of BA dynamics at whole body level, with sufficient accuracy.

Future improvements of the model will include differentiation in various BAs (primary and secondary) and their metabolites. This extension of the current PBBA model is important since BAs are continuously transformed and may accumulate differentially in various tissues all over the body. The prediction of such shifts in BA composition in specific tissues like the liver based on simpler blood samples could also be a fine-tuned biomarker for the assessment of the different diseases as well as cholestasis. For such a differentiated BA pool the necessary metabolisation steps which are catalysed by the intestinal microbiome need to be integrated. The tools for the vertical integration of metabolic network models into PBPK models already exist and can directly be added to the current PBBA model (Krauss et al., 2012; Cordes et al., 2018).

There is also growing interest in the role of BAs as mediators and signalling molecules within systemic circulation at the whole-body level. For example, it has been shown that BAs play an essential role in the activation of cellular receptors like GPBAR1 (G protein-coupled bile acid receptor 1) or FXR- α (farnesoid X receptor) (Hylemon et al., 2009). Likewise, BAs have been shown to regulate intracellular pathways such as insulin signalling in the liver or the intestinal tract as well as energy metabolism in brown adipose tissue (Watanabe et al., 2006). Consequently, a therapeutic administration of BAs may help to treat metabolic diseases through fine-tuning of metabolic control (Broeders et al., 2015). There are also indications that BAs influence energy metabolism beyond enterohepatic circulation in the central nervous system through direct or indirect pathways (Mertens et al., 2017). Metabolomics studies have identified bile acids as biomarkers for various pathologies such as hepatic impairment in polycystic kidney disease (Brock et al., 2018), gestational diabetes (Gao et al., 2016), hepatitis B-induced cirrhosis (Wang et al., 2016), Alzheimer’s disease (Marksteiner et al., 2018). Also in this regard could an extended PBBA model be of use in the future to mechanistically describe and explain BA disposition in specific tissues as well as the underlying multi-tissue interplay.

Further modifications of the presented PBBA model could include circadian BA synthesis or gallbladder emptying overnight, which have been neglected in the present version of the model. Therefore, the simulated nightly BA profiles are not as reliable as of now. Additionally, different meal compositions could be considered to specifically trigger different responses. This could also include the effect of change in lifestyle on the composition of the intestinal microbiome and subsequent changes in BA composition (Wahlström et al., 2016). Furthermore, specific preclinical *in vitro* data can be integrated and used for *in vitro-in vivo* translation of omics data (Kuepfer et al., 2017). This will ideally involve time series of omics data which could be contextualised in the model to describe the adaption of bile acid metabolism toward repeated drug administration or to track specific pathogeneses. The clinical cases shown in this work, however, illustrate that the current model can already be applied to analyses of clinical relevance. Another extension of the current PBBA model could be its translation to preclinical animal species to mechanistically support the analysis of targeted experimental measurements such as two-photon imaging data (Reif et al., 2017) or to investigate physiological phenomena at systems level such as bile infarct formation (Ghallab et al., 2019). We therefore strongly believe that the presented model provides an important platform for model-based analyses of BA metabolism in the future.

## Data Availability

The raw data supporting the conclusions of this manuscript will be made available by the authors, without undue reservation, to any qualified researcher.

## Author Contributions

VB and LK: writing the original draft. VB, HC, CT, JC, UN, LB, and LK: writing the review and editing. VB and LK: project design. VB: analysis and implementation.

## Conflict of Interest Statement

LK is employee of Bayer AG.

The remaining authors declare that the research was conducted in the absence of any commercial or financial relationships that could be construed as a potential conflict of interest.

## Footnotes

1 https://github.com/Open-Systems-Pharmacology

2 https://github.com/ankitrohatgi/WebPlotDigitizer
