# Supplementary material for: A Physiology-Based Model of Human Bile Acid Metabolism for Predicting Bile Acid Tissue Levels After Drug Administration in Healthy Subjects and BRIC Type 2 Patients
Source: Front Physiol. 2019 Sep 27;10:1192. doi: 10.3389/fphys.2019.01192 (PMC6777137; doi:10.3389/fphys.2019.01192)
Supplement: Supplementary file 2 [file Data_Sheet_2.docx]

Supplementary Material

# Supplementary Data

The MoBi model file contains the simulations of a healthy and a BRIC2 individual as well as the both with concomitant cyclosporine A dosing. Simulations can be exported as report file where all the model formulae are accessible. Simulations can also be exported to xml format and then simulated with the Matlab or R toolbox. Documentation how to do this is available on the OSPS github repository.

The provided population csv sheet defines the 1,000 individuals of the virtual healthy population. In Matlab, this can be used for the population simulation by initialising the individuals and running the simulation to steady state.

| Population.csv | csv file with the parameters defining the individuals of the virtual population |
| --- | --- |
| PBBA.mbp3 | MoBi model file with the simulations of the healthy, the BRIC2 individuals, and the two combined with CsA |

# Model building

The basic PBBA model for GCDCA is a PBPK model including four active transport
processes (BSEP, NTCP, ASBT and OSTα) (Figure 2, *see workflow below*). Physico-chemical parameters of the bile salt GCDCA (Table 1) as well as biometry of the individual (Table 2) can be specified in the GUI of PK-Sim. Likewise, transporter localisation in different tissues (organ subcompartments as well as apical or basolateral position), directionality (influx or efflux) and rate laws (first order or Michaelis-Menten kinetics) can be directly selected through the GUI. Note that the transporter equations are automatically generated in the PBPK model.

All transporter equations have the following structure:

$${transport}_{i}(t)=k_{cat,i}\cdot E_{0}\cdot\frac{c_{GCDCA}(t)}{c_{GCDCA}\left( t \right)+K_{m,i}}$$

For each transporter *i*, the catalytic rate constant $k_{cat,i}$, the protein concentration $E_{0}$ and the Michaelis constant $K_{m,i}$ are needed (Table 3). The concentration $c_{GCDCA}$ in the source compartment then determines the transport rate of transporter *i* as a function of time $t$.

The PBPK model of GCDCA requires specification of meal events to trigger gall bladder emptying (*see workflow below*). Fill-up of the gallbladder is balanced in PK-Sim by biliary secretion. In the PBBA model this occurs via the BSEP transporter as specified above. Discontinuous release of bile from the gallbladder is specified in PK-Sim by providing the start times of the bile ejection (equal to the times of meal intake) and the duration of the ejection event given by the *refilling time* (Table 2).

The ejection rate of bile ejection is specified as follows

$$ejection(t)=M(t)\cdot\frac{ln(2)}{T_{Emptying half-life}}$$

where $M(t)$ is the amount of bile acids [µmol] in the gallbladder and emptying half-life $T_{Emptying half-life}$ is the half life of GCDCA in the gallbladder during the emptying event.

The PBPK model including the four parametrised transporter reactions is then exported to MoBi to add the synthesis reaction of GCDCA

$${synthesis}_{GCDCA}=PARAM\_G\_CDCA\_synthesis\_rate$$

with $PARAM\_G\_CDCA\_synthesis\_rate$ =0.78161 µmol/min.

The steps of the workflow for model building are described in detail below.

The model is now complete and ready for simulation (see Supplementary Material for MoBi model file).

Note, that all equations of the model including the parameter values can be assessed in an ASCII-file by right clicking the simulation in the Mobi-mbp3 file and selecting “Create Simulation Report”.

**Build basic PBPK model for GCDCA in PK-Sim**

Add building block “Individual” with the following specifications

1a. Biometrics: Use default settings (European male, 30 years, 73 kg, 176 cm)
1b. Anatomy & Physiology: Use default settings
1c. Specify active processes in the expression tab:

- - - Add metabolizing enzyme:

| - Name | - CYP7A1 |
| --- | --- |
| - Rel. expression (Liver Periportal) | - 1 |

- - - Add transporters (4 in total):

| - Transporter | - Additional steps | - Values |
| --- | --- | --- |
| - BSEP/ABCB11 - (RT-PCR from PK-Sim database) | - Rel. expression (Gonads) | - 0 |
| - NTCP/SLC10A1 - (RT-PCR from PK-Sim database) | - Rel. expression - (All but liver) | - 0 |
| - ASBT/SLC10A2 | - Transport type | - Influx |
|  | - Rel. expression (Upper Ileum) | - 1 |
|  | - Rel. expression (Lower Ileum) | - 1 |
| - OSTalpha | - Transport type | - Efflux |
|  | - Rel. Expression (Mucosa Small Intestine) | - 1 |
|  | - Rel. Expression (Mucosa Large Instestine) | - 0.75 |

2. Add building block “Compound” with following specifications:

- Provide basic physico-chemistry information of G-CDCA

| - Name | - G-CDCA |
| --- | --- |
| - Lipophilicity | - 2.12 |
| - Fraction unbound/ binding partner | - 0.01/Albumin |
| - Molecular weight | - 449.62 |
| - pKa | - 3.77 (acidic) |
| - Solubility | - 100,000 mg/l |

- Specify associated ADME proteins (choose Michaelis-Menten kinetics for all)

| - Protein | - kcat [1/min] | - Km [µmol/l] |
| --- | --- | --- |
| - CYP7A1 | - Placeholder | - Placeholder |
| - BSEP/ABCB11 | - 300 | - 5 |
| - NTCP/SLC10A1 | - 125 | - 1 |
| - ASBT/SLC10A2 | - 5 | - 0.5 |
| - OSTalpha | - 9000 | - 7.5 |

3. Add building block “Administration”:

- Create an arbitrary Administration protocol for G-CDCA;
  (this has to be done to be able to create a simulation in PK-Sim and will be deleted afterwards)

4. Add building block “Event”:

- Create an event of the type Meal: Standard (Human)

5. Create a simulation from the building blocks

5a. Map the processes
5b. Add the meal event in the pattern 0h - 4h - 8h per 24hours
5c. Export (send) this simulation to MoBi

**Extend PBPK model for G-CDCA in MoBi**

1. Create a new reaction:

1a. In the “Properties” tab, specify

| - Name | - G-CDCA synthesis |
| --- | --- |
| - Formula type | - Formula (an explicit formula) |
| - Formula name | - Add Formula > Name: FORMULA G-CDCA synthesis |
| - dN/dt | - PARAM_G_CDCA_synthesis_rate |

1b. Add a new parameter in the “Parameters” tab to quantify the synthesis rate of GCDCA

| Name | PARAM G-CDCA synthesis |
| --- | --- |
| Parameter type | Global |
| Dimension | Amount per time |
| Group | MoBi |
| Formula type | Constant (a single numeric value) |
| Value | 0.78161 |

1c. Specify the container criteria in the “Container criteria” tab

| - Match tag condition | - Periportal |
| --- | --- |
| - Match tag condition | - Intracellular |

2. Delete the administration protocol:

- - Change all the parameters specified in the manuscript to the corresponding values

3. Simulate

3a. Export the simulation to xml

3b. Run the simulation to steady state (e.g. 600h) and export the G-CDCA concentrations in each
 compartment to an xls file

3c. Import the xls into the Molecule Start Values and update the simulation

# Supplementary Figures


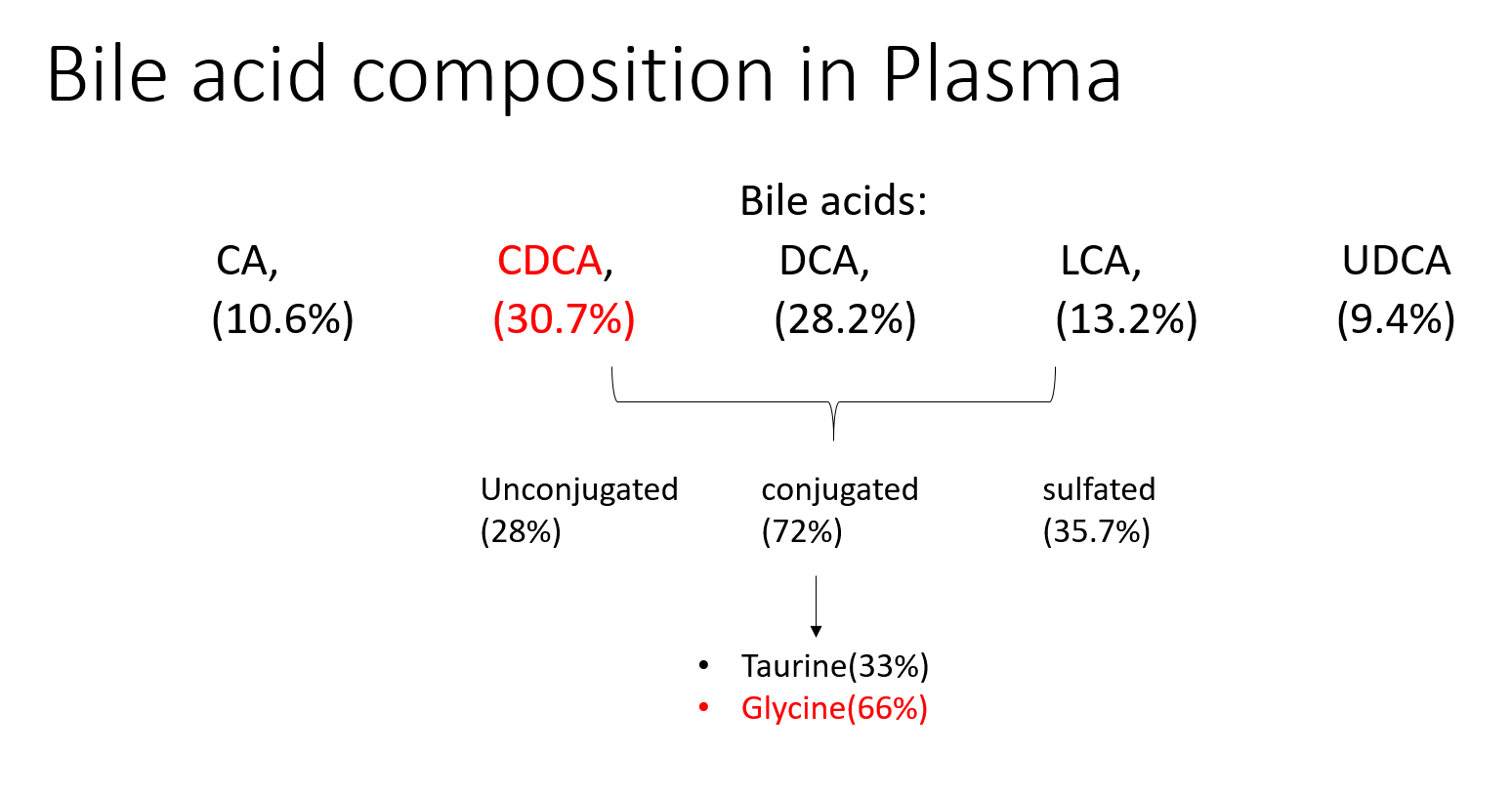


Supplementary Figure S1. Bile acid composition in blood plasma.

**
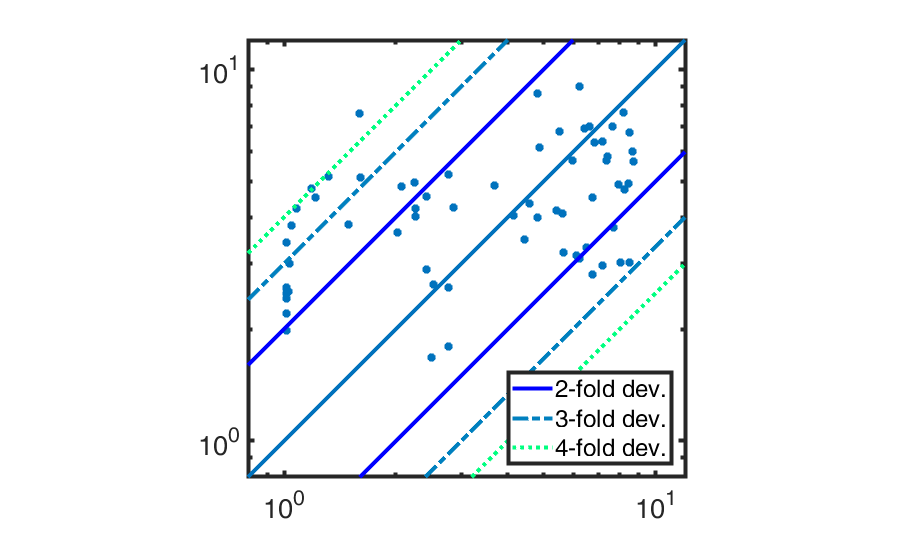
**

**Supplementary Figure S2**. Predicted vs. observed plot with k-fold deviation.

# Supplementary Tables

Table S1 Scaling for heterogenous bile acid measurements.

| **Study** | **Scaling factor** |
| --- | --- |
| (Hepner and Demers, 1977) | 2.03 |
| (Schalm et al., 1978) | 1.68 |
| (Angelin and Björkhem, 1977) | 0.66 |
| (Bathena et al., 2013) | - |
| (Galeazzi et al., 1980) | 2.57 |
| (Gälman et al., 2005) | 2.57 |
| (Angelin and Björkhem, 1977) | 0.66 |
| (Ponz de Leon et al., 1978) | 1.68 |
| (Setchell et al., 1997) | 2.57 |
| (Salemans, J. M. J. I. et al., 2009) | 1 |
